# The Influence of Work Values on Nursing Career Intentions: A Study of Multiple Mediating Factors

**DOI:** 10.1155/jonm/2966855

**Published:** 2026-09-06

**Authors:** Chung-I. Lin, Chih-Feng Lai, Wei-Cheng Chien, Ming-Hsueh Tsai, Ming-Chuan Hsieh, Ming-Chi Wu, Chin-Hsiang Yen

**Affiliations:** ^1^ National Academy for Educational Research, New Taipei City, Taiwan; ^2^ Department of Education, National Taichung University of Education, Taichung, Taiwan, ntcu.edu.tw; ^3^ Department of Education and Learning Technology, National Tsing Hua University, Hsinchu, Taiwan, nthu.edu.tw; ^4^ Jenteh Junior College of Medicine, Nursing and Management, Miaoli, Taiwan, jente.edu.tw

**Keywords:** burnout, career intention, job satisfaction, theory of planned behavior, work values

## Abstract

In light of the global shortage in the nursing workforce, understanding the factors associated with career intention among nursing students and registered nurses has become increasingly important for nursing education and workforce retention strategies. Grounded in the theory of planned behavior (TPB), this study developed an extended TPB model incorporating work values and supplementary workplace psychological variables to examine the psychological mechanisms associated with nursing career intention across different professional stages. A cross‐sectional survey design was employed, in which nursing students from Taiwan’s technical and vocational institutions, as well as practicing registered nurses, were invited via institutional email channels to complete an online questionnaire. A total of 2137 valid responses were collected, including 1068 from nursing students and 1069 from registered nurses. Structural equation modeling (SEM) was used for data analysis. The results indicated that partial mediation models demonstrated significantly better fit than full mediation models in both groups. Among nursing students, work values were directly associated with career intention and were also indirectly associated with career intention through attitude, subjective norms, and perceived behavioral control. Among registered nurses, work values demonstrated both direct and indirect relationships with career intention. The indirect relationship was primarily observed through perceived behavioral control, whereas burnout showed a statistically significant but relatively small indirect effect. Job satisfaction was positively associated with work values, although its indirect pathway to career intention was not statistically significant in the final partial mediation model. For both groups, perceived behavioral control demonstrated the strongest relationship with career intention. The findings suggest that work values may function not only as antecedents of TPB‐related beliefs but also as broader motivational orientations associated with nursing career intention across both educational and workplace contexts. Strengthening value‐oriented education and workplace support may contribute to more favorable professional motivation and psychological adaptation among nursing personnel.

## 1. Introduction

In the context of global population aging and increasing public health challenges, shortages in the nursing workforce and high turnover rates have become pressing concerns for healthcare systems worldwide. Clinical nurses frequently face heavy workloads, emotional exhaustion, and demanding workplace conditions, which contribute to burnout and reduced willingness to remain in the profession [1, 3]. Enhancing career intention and promoting long‐term retention among nursing personnel have therefore become important issues in nursing education and healthcare management. Understanding the psychological and workplace‐related factors associated with nursing career intention may provide valuable implications for workforce sustainability and professional development. Among the factors associated with career intention, work values have been recognized as an important antecedent influencing occupational attitudes and behavioral tendencies. Work values refer to individuals’ subjective beliefs and preferences regarding the meaning and functions of work, including dimensions such as financial rewards, social contribution, job stability, self‐fulfillment, and professional achievement [4–6]. Previous studies have shown that when individuals perceive stronger alignment between their personal values and occupational characteristics, they tend to report higher occupational commitment, stronger professional identity, and more positive work‐related outcomes [4, 7]. In nursing contexts, work values may be particularly important because nursing professionals often encounter emotionally demanding and high‐stress environments. Stronger work values may help individuals interpret workplace experiences more positively and maintain motivation to remain in the profession [4, 6]. In addition, previous studies have suggested that work values may demonstrate direct relationships with nursing career intention and employment intention [8, 9], indicating that work values may function not only as antecedents of psychological beliefs but also as broader motivational orientations associated with occupational commitment and career choice.

The theory of planned behavior (TPB), proposed by Ajzen [10], has been widely applied to explain behavioral intentions and occupational decision‐making processes. According to the TPB, behavioral intention is primarily determined by three core psychological constructs: attitude, subjective norms, and perceived behavioral control. Attitude refers to individuals’ positive or negative evaluations of performing a behavior; subjective norms refer to perceived social expectations from significant others; and perceived behavioral control reflects individuals’ beliefs regarding their capability and control over performing the behavior [10]. Previous studies have demonstrated the applicability of TPB in explaining nurses’ behavioral intentions, including turnover intention, clinical decision‐making, and technology adoption behaviors [11–13]. Moreover, prior research has reported that personal values are significantly associated with TPB‐related beliefs and behavioral intentions [14–16]. Accordingly, work values may influence nursing career intention both indirectly through TPB constructs and directly as broader motivational orientations associated with professional commitment and occupational intention.

In addition to the TPB framework, workplace psychological factors may also play an important role in shaping nurses’ career intention, particularly among employed clinical nurses. Burnout has frequently been associated with emotional exhaustion, occupational stress, and turnover intention in nursing environments [17, 18]. Similarly, job satisfaction has consistently been identified as a factor associated with nurses’ retention intention and professional commitment [1, 19–21]. Previous studies grounded in the Job Demand–Control Model [22] and Herzberg’s Two‐Factor Theory [23] have highlighted the importance of workplace conditions and psychological experiences in influencing occupational outcomes. Rather than directly testing the core propositions of these theories, the present study incorporates burnout and job satisfaction as supplementary workplace psychological variables to extend the TPB framework within nursing settings. This approach allows for a more comprehensive understanding of how workplace experiences may be associated with career intention among registered nurses.

Previous studies have shown that work values positively relate to TPB constructs [14–16], whereas burnout and job satisfaction are associated with career intention among the nursing personnel [4, 17, 18]. In addition, prior studies have reported direct relationships between work values and nursing career intention or employment intention [8, 9]. However, relatively limited research has simultaneously examined both the direct and indirect relationships of work values with career intention across nursing students and registered nurses within a unified analytical framework. Nursing students and registered nurses represent different stages of professional development, and the psychological mechanisms associated with career intention may differ between educational and clinical contexts. Therefore, examining both groups simultaneously may provide additional insights into the role of work values and workplace psychological factors across different career stages in nursing.

Accordingly, this study develops an extended TPB model to investigate how work values are associated with nursing career intention both directly and indirectly through TPB constructs (attitude, subjective norms, and perceived behavioral control), while further examining whether burnout and job satisfaction serve as supplementary mediating variables among registered nurses. By comparing nursing students and registered nurses as parallel samples, this study aims to provide supplementary evidence regarding the psychological processes associated with career intention across different professional stages in nursing. The specific research questions are as follows:

1. Do work values directly and indirectly relate to career intention among nursing personnel through attitude, subjective norms, and perceived behavioral control?
2. Among registered nurses, do burnout and job satisfaction serve as supplementary mediating variables in the relationship between work values and career intention?
3. Through a comparative analysis of nursing students and registered nurses, how can we better understand the psychological mechanisms associated with nursing career intention across different professional stages?

## 2. Materials and Methods

### 2.1. Study Design and Participants

**FIGURE 1:**
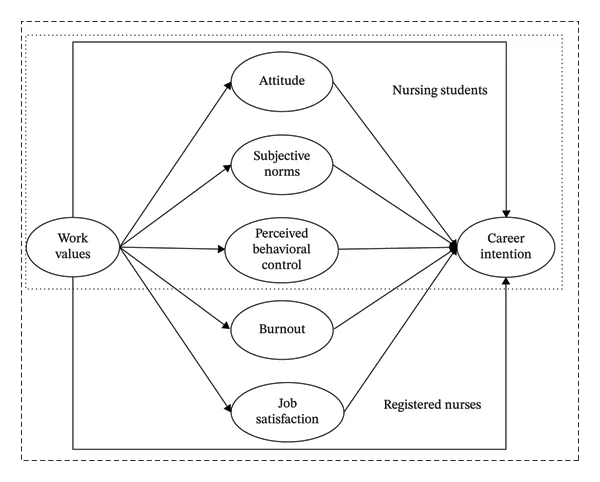
Research framework.

This study was grounded in the TPB and employed structural equation modeling (SEM) to investigate the psychological mechanisms associated with career intention among nursing students and registered nurses. According to the TPB, behavioral intention is primarily associated with three core constructs: attitude, subjective norms, and perceived behavioral control. Based on this framework, the present study positioned work values as an important antecedent variable and examined both its direct and indirect relationships with career intention through the three TPB constructs. In addition, because workplace psychological experiences may play a particularly important role among employed clinical nurses, burnout and job satisfaction were incorporated into the registered nurse model as supplementary workplace psychological variables. Rather than directly testing the core propositions of the Job Demand–Control Model or Herzberg’s Two‐Factor Theory, these variables were included to extend the TPB framework within nursing contexts and provide a more comprehensive understanding of how workplace experiences may be associated with nurses’ intention to remain in the profession. To further examine the mediation structure, alternative structural models, including direct‐effect, full mediation, and partial mediation models, were compared. Separate SEM models were developed and tested for nursing students and registered nurses. The analyses examined direct, indirect, and total effects among variables to better understand the psychological processes associated with nursing career intention across different professional stages. The overall research framework is illustrated in Figure 1.

A cross‐sectional survey design was adopted, and data were collected via online questionnaires from March to May 2025. This study was reported in accordance with the Strengthening the Reporting of Observational Studies in Epidemiology (STROBE) statement. The study targeted nursing students from junior colleges and technological universities in Taiwan, as well as licensed clinical nurses currently employed in healthcare settings. Eligible nursing students were those currently enrolled in nursing‐related academic programs in Taiwan. Eligible registered nurses were those currently employed in healthcare institutions in Taiwan and holding a valid nursing license. Participation was voluntary for both groups. The online survey system required all questionnaire items to be completed before submission. According to national statistics for the 2025–2026 academic year, there were 47 higher education institutions offering nursing‐related programs in Taiwan, with a total student population of approximately 49,141. In addition, there were approximately 198,526 actively practicing registered nurses nationwide, representing the primary professional population of interest. Based on the sample size determination formula proposed by Krejcie and Morgan [24], at a 0.05 significance level with a ±3% sampling error, the minimum required sample sizes were 1044 for nursing students and 1061 for registered nurses. A nationwide institution‐mediated recruitment approach was used. Official invitation letters were sent to nursing‐related academic departments and healthcare institutions across Taiwan, and these institutions were asked to forward the online survey invitation to eligible nursing students or registered nurses via email. This approach enabled broad coverage across multiple educational institutions and healthcare settings. All responses were anonymous, and the data were used solely for academic research purposes. A total of 1068 valid questionnaires were collected from nursing students and 1069 from registered nurses, both exceeding the respective minimum required sample sizes. As the invitations were distributed through institutional email systems, the exact number of recipients could not be determined. Therefore, a precise response rate could not be calculated, which should be considered a limitation of the study. Nevertheless, the sample size obtained was relatively large and exceeded the minimum required sample sizes, supporting the statistical analyses conducted in this study.

### 2.2. Measuring Tools

This study used seven scales, each comprising four items. All items were rated on a five‐point Likert scale, ranging from 1 (strongly disagree) to 5 (strongly agree). The work value items were adapted from prior studies on nurses’ work values, particularly the conceptual framework of the Nurses’ Work Values Scale (NWVS) developed by Hara et al., covering intrinsic, extrinsic, social, and prestige‐related work values [6]. The scales for attitude, subjective norms, perceived behavioral control, and career intention were developed with reference to the existing literature [10, 25]. The burnout scale was developed based on previous research [20, 25], while the job satisfaction scale was adapted from Zhao et al. [19]. In addition, five experts and scholars with professional backgrounds in nursing education and clinical nursing were invited to review and refine the self‐developed questionnaire items to ensure adequate content validity and contextual appropriateness.

Cronbach’s *α* values for nursing students ranged from 0.79 to 0.91, while those for registered nurses ranged from 0.80 to 0.91, indicating satisfactory internal consistency reliability. In addition, confirmatory factor analysis (CFA) was conducted separately for the nursing student and registered nurse samples to evaluate the measurement models. The measurement model for nursing students demonstrated an acceptable fit to the data (*X* ^2^ = 1106.20, *p* < 0.001; RMSEA = 0.07; CFI = 0.93; TLI = 0.91; SRMR = 0.04). Similarly, the measurement model for registered nurses also demonstrated an acceptable fit (*X* ^2^ = 1737.94, *p* < 0.001; RMSEA = 0.06; CFI = 0.92; TLI = 0.91; SRMR = 0.04).

Convergent validity was assessed using standardized factor loadings, composite reliability (CR), and average variance extracted (AVE). For nursing students, the CR values ranged from 0.80 to 0.91, and the AVE values ranged from 0.50 to 0.72. For registered nurses, the CR values ranged from 0.81 to 0.91, and the AVE values ranged from 0.52 to 0.72, supporting acceptable convergent validity. Discriminant validity was evaluated using the Fornell–Larcker criterion. Overall, the square roots of the AVE values were comparable to or exceeded the inter‐construct correlations, indicating acceptable discriminant validity. In addition, discriminant validity was further examined using the heterotrait–monotrait (HTMT) ratio. All HTMT values were below the recommended threshold of 0.85, further supporting satisfactory construct distinctiveness among the latent variables. In addition, all standardized factor loadings were statistically significant and exceeded the recommended threshold, further supporting satisfactory measurement quality.

### 2.3. Data Analysis

Prior to hypothesis testing, several statistical assumption checks were conducted. Univariate normality was examined using skewness and kurtosis statistics. For the nursing student sample, skewness values ranged from −1.04 to −0.20, and kurtosis values ranged from −0.49 to 0.89. For the registered nurse sample, skewness values ranged from −0.75 to −0.05, and kurtosis values ranged from −0.86 to 0.54, indicating acceptable normality of the observed variables. Linearity among the latent variables was examined through correlation analyses and scatterplot inspection, while potential outliers were assessed using Mahalanobis distance statistics. In addition, multicollinearity was evaluated using variance inflation factor (VIF) values. The VIF values ranged from 1.67 to 4.66, which were below the recommended threshold of 5.0, indicating no serious multicollinearity concerns.

SPSS was used for descriptive statistical analyses and preliminary data screening, while Mplus was employed for CFA and SEM. As described in Section 2.2, CFA was conducted to evaluate the measurement models and establish construct validity prior to structural model testing. SEM was subsequently performed using the robust maximum likelihood estimator (MLR) estimator in Mplus to test the hypothesized relationships and mediation effects. Indirect effects were primarily estimated using the product‐of‐coefficients approach with the delta method. Given the limitations of the delta method in assuming normal distributions of indirect effects, bootstrap confidence intervals with 5000 resamples were additionally conducted to enhance the robustness of the mediation analysis. Statistical significance was determined at the 0.05 level.

### 2.4. Ethical Considerations

This study was conducted in accordance with the ethical review procedures of the principal investigator’s affiliated institution. Prior to data collection, the study protocol was reviewed by the institutional research review committee and was determined to be exempt from formal ethical review. The exemption was granted because the study involved an anonymous online survey, did not collect personally identifiable information, posed no more than minimal risk to participants, and involved voluntary participation. Prior to survey participation, all participants were provided with an online consent form explaining the study’s objectives, procedures, data usage, and confidentiality policies. Only those who gave informed consent were allowed to proceed with the survey. All responses were collected anonymously, with no personally identifiable information involved. The study strictly complied with Taiwan’s research ethics regulations and personal data protection laws. Participation was entirely voluntary, and respondents could withdraw from the study at any time without any negative consequences.

## 3. Results and Discussion

### 3.1. Participants’ Characteristics

Table 1 presents the one‐way ANOVA results on career intention among nursing students. A total of 1068 participants were surveyed, of whom 90.36% were female and 9.64% male. In terms of age, the largest group was 19–20 years old (40.54%), followed by 21–23 years old (26.03%), under 18 years old (23.69%), and over 24 years old (9.74%). Regarding school type, 48.03% attended private junior colleges and technological universities, followed by 30.81% in private general universities, 11.42% in public general universities, and 9.74% in public junior colleges and technological universities. Concerning relevant work or volunteer experience in nursing, 57.12% reported having such experience, while 42.88% did not. Regarding postgraduation plans, 35.30% intended to pursue further education, 44.57% were undecided, and 20.13% did not plan to continue studying.

**TABLE 1:** ANOVA results on background and career intention of nursing students.

	*N*	%	Mean	SD	*F*	*η* ^2^	Post hoc tests
Age					6.27^∗∗∗^	0.017	24 years↑> 18 years↓, 19–20 years, and 21–23 years
1.18 years↓	253	23.69	3.63	0.88			
2.19–20 years	433	40.54	3.50	0.92			
3.21–23 years	278	26.03	3.62	0.84			
4.24 years↑	104	9.74	3.90	0.78			

Gender					4.14^∗^	0.008	Female > Male
1. Female	965	90.36	3.62	0.86			
2. Male	103	9.64	3.60	1.05			

School Type					1.88	0.005	—
1. Public general universities	122	11.42	3.47	0.97			
2. Private general universities	329	30.81	3.56	0.85			
3. Public junior colleges and technological universities	104	9.74	3.62	0.92			
4. Private junior colleges and technological universities	513	48.03	3.66	0.87			

Relevant Nursing Work or Volunteer Experience					9.17^∗∗^	0.009	Experience > No experience
1. No	458	42.88	3.51	0.91			
2. Yes	610	57.12	3.67	0.86			

Planned to Pursue Further Education					40.79^∗∗∗^	0.071	Planned > Undecided > No plan
1. No	215	20.13	3.22	1.02			
2. Yes	377	35.30	3.87	0.83			
3. Undecided	476	44.57	3.56	0.79			

^∗∗∗^ *p* < 0.001.

^∗∗^ *p* < 0.01.

^∗^ *p* < 0.05.

The ANOVA results showed significant differences in career intention across age (*F* = 6.27, *p* < 0.001, *η* ^2^ = 0.017), gender (*F* = 4.14, *p* < 0.05, *η* ^2^ = 0.008), relevant nursing work or volunteer experience (*F* = 9.17, *p* < 0.01, *η* ^2^ = 0.009), and postgraduation plans (*F* = 40.79, *p* < 0.001, *η* ^2^ = 0.071). However, the effect sizes for gender and relevant nursing work or volunteer experience were relatively small. Post hoc comparisons indicated that students aged 24 years or above (*M* = 3.90, SD = 0.78) reported significantly higher career intention than those aged 18 years or below (*M* = 3.63, SD = 0.88), 19–20 years (*M* = 3.50, SD = 0.92), and 21–23 years (*M* = 3.62, SD = 0.84). Female students (*M* = 3.62, SD = 0.86) reported slightly higher career intention than male students (*M* = 3.60, SD = 1.05), although the observed effect size was negligible. Students with relevant nursing work or volunteer experience (*M* = 3.67, SD = 0.86) also reported slightly higher career intention than those without such experience (*M* = 3.51, SD = 0.91), although the observed effect size was small. In addition, students who planned to pursue further education (*M* = 3.87, SD = 0.83) demonstrated comparatively stronger career intention than those who had no plans for further education (*M* = 3.22, SD = 1.02) or were undecided (*M* = 3.56, SD = 0.79), with a moderate effect size.

The ANOVA results for registered nurses’ career intention are presented in Table 2, based on 1069 valid responses. Of these, 94.38% were female and 5.62% male. Most were between 40 and 49 years old (31.34%), followed by 30–39 years (27.78%), 20–29 years (21.61%), 50–59 years (17.59%), and over 60 years (1.68%). Regarding educational attainment, 65.00% held a bachelor’s degree, followed by 19.80% with a master’s degree, 13.80% with an associate’s degree from junior colleges and technological universities, and 1.30% with a doctoral degree. In terms of years of service, 47.15% had over 16 years of experience, followed by 18.05% with 11–15 years, 16.65% with 6–10 years, 15.15% with 1–5 years, and 2.99% with less than 1 year. Regarding weekly working hours, most worked 41–60 h (65.39%), followed by 21–40 h (29.56%), more than 60 h (4.40%), and less than 20 h (0.65%).

**TABLE 2:** ANOVA results on background and career intention of registered nurses.

	*N*	%	Mean	SD	*F*	*η* ^2^	Post hoc tests
Age					13.47^∗∗∗^	0.048	50–59 y > 40–49 y > 30–39 y > 20–29 y; 60 y↑> 20–29 y
1. 20–29 y	231	21.61	3.04	0.88			
2. 30–39 y	297	27.78	3.23	0.99			
3. 40–49 y	335	31.34	3.40	0.81			
4. 50–59 y	188	17.59	3.64	0.87			
5. 60 years↑	18	1.68	3.57	0.77			

Gender					4.63^∗∗^	0.009	Female > Male
1. Female	1009	94.38	3.34	0.90			
2. Male	60	5.62	2.97	1.04			

Education level					8.49^∗∗∗^	0.023	Master > Bachelor, Associate
1. Associate’s degree	148	13.80	3.20	0.84			
2. Bachelor’s degree	695	65.00	3.26	0.90			
3. Master’s degree	212	19.80	3.59	0.93			
4. Doctoral degree	14	1.30	3.50	1.01			

Years of experience					12.62^∗∗∗^	0.045	16 years↑ > 1 years↓, 1–5 years, 6–10 years
1. 1 years↓	32	2.99	2.98	0.85			
2. 1–5 years	162	15.15	3.04	0.91			
3. 6–10 years	178	16.65	3.11	0.98			
4. 11–15 years	193	18.05	3.34	0.90			
5. 16 years↑	504	47.15	3.50	0.85			

Weekly working hours					3.72^∗^	0.010	21–40 hours > 41–60 hours
1. 20 h↓	7	0.65	3.29	0.67			
2. 21–40 h	316	29.56	3.46	0.91			
3. 41–60 h	699	65.39	3.25	0.90			
4. 61 hours↑	47	4.40	3.36	0.99			

^∗∗∗^ *p* < 0.001.

^∗∗^ *p* < 0.01.

^∗^ *p* < 0.05.

The ANOVA results revealed significant differences in career intention across age (*F* = 13.47, *p* < 0.001, *η* ^2^ = 0.048), gender (*F* = 4.63, *p* < 0.01, *η* ^2^ = 0.009), education level (*F* = 8.49, *p* < 0.001, *η* ^2^ = 0.023), years of experience (*F* = 12.62, *p* < 0.001, *η* ^2^ = 0.045), and weekly working hours (*F* = 3.72, *p* < 0.05, *η* ^2^ = 0.010). However, the effect sizes for gender and weekly working hours were relatively small. Post hoc comparisons showed that nurses aged 50–59 years (*M* = 3.64, SD = 0.87) reported significantly higher career intention than those aged 40–49 years (*M* = 3.40, SD = 0.81), 30–39 years (*M* = 3.23, SD = 0.99), and 20–29 years (*M* = 3.04, SD = 0.88). Nurses aged 60 years or above (*M* = 3.57, SD = 0.77) also reported higher career intention than those aged 20–29 years. Nurses with a master’s degree (*M* = 3.59, SD = 0.93) reported significantly higher career intention than those with a bachelor’s degree (*M* = 3.26, SD = 0.90) or an associate’s degree (*M* = 3.20, SD = 0.84). In addition, nurses with more than 16 years of experience (*M* = 3.50, SD = 0.85) demonstrated significantly higher career intention than those with less than 1 year (*M* = 2.98, SD = 0.85), 1–5 years (*M* = 3.04, SD = 0.91), or 6–10 years of experience (*M* = 3.11, SD = 0.98). Regarding weekly working hours, nurses working 21–40 h per week (*M* = 3.46, SD = 0.91) reported significantly higher career intention than those working 41–60 h per week (*M* = 3.25, SD = 0.90). Female nurses (*M* = 3.34, SD = 0.90) also reported slightly higher career intention than male nurses (*M* = 2.97, SD = 1.04), although the observed effect size was small.

### 3.2. Nursing Students’ Model Results

**FIGURE 2:**
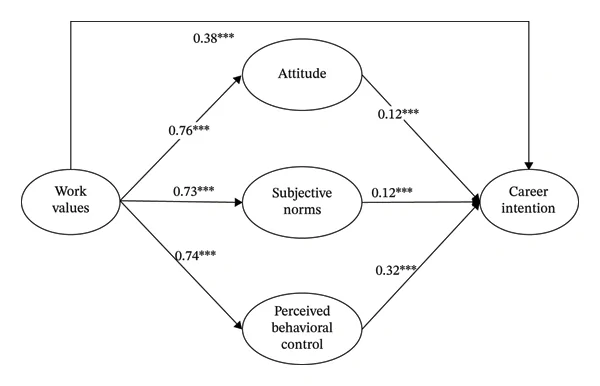
Standardized path coefficients of the nursing student model.

To further examine the mediation structure, alternative structural models were compared. First, a direct‐effect model examining the relationship between work values and career intention was tested. Although the direct path from work values to career intention was statistically significant (*β* = 0.78, *p* < 0.001), the RMSEA was not fully satisfactory. Therefore, alternative full and partial mediation models were subsequently compared. The full mediation model and the partial mediation model were compared using *χ* ^2^ difference testing. The partial mediation model demonstrated a significantly better fit than the full mediation model (Δ*χ* ^2^ = 33.05, Δ*df* = 1, *p* < 0.001). In addition, the partial mediation model showed slightly improved fit indices, including higher CFI and TLI values and lower RMSEA and SRMR values. Specifically, CFI increased from 0.927 to 0.930, TLI increased from 0.916 to 0.918, RMSEA decreased from 0.075 to 0.074, and SRMR decreased from 0.045 to 0.044. Therefore, the partial mediation model was retained as the final model for nursing students. The final partial mediation model demonstrated acceptable fit indices (*χ* ^2^ = 1128.37, *df* = 163, *p* < 0.001; CFI = 0.93; TLI = 0.92; RMSEA = 0.07; SRMR = 0.04). The standardized path coefficients are illustrated in Figure 2, while the total, direct, and indirect effects are summarized in Table 3.

**TABLE 3:** Total, direct, and indirect effects of the nursing student model.

	Estimate	S.E.	Est./S.E.
Direct effects			
Work values ⟶ attitude	0.76	0.02	42.66^∗∗∗^
Work values ⟶ subjective norms	0.73	0.02	35.14^∗∗∗^
Work values ⟶ perceived behavioral control	0.74	0.02	41.06^∗∗∗^
Work values ⟶ career intention	0.38	0.07	5.48^∗∗∗^
Attitude ⟶ career intention	0.12	0.04	2.80^∗∗^
Subjective norms ⟶ career intention	0.12	0.04	3.01^∗∗^
Perceived behavioral control ⟶ career intention	0.32	0.04	8.69^∗∗∗^
Indirect effects			
Work values ⟶ attitude ⟶ career intention	0.09	0.05	1.98^∗^
Work values ⟶ subjective norms ⟶ career intention	0.09	0.04	2.27^∗^
Work values ⟶ perceived behavioral control ⟶ career intention	0.23	0.04	6.27^∗∗∗^
Total effect	0.79	0.02	35.88^∗∗∗^

^∗∗∗^ *p* < 0.001.

^∗∗^ *p* < 0.01.

^∗^ *p* < 0.05.

Regarding direct effects, work values had significant positive relationships with attitude (*β* = 0.76, *p* < 0.001), subjective norms (*β* = 0.73, *p* < 0.001), perceived behavioral control (*β* = 0.74, *p* < 0.001), and career intention (*β* = 0.38, *p* < 0.001). Attitude (*β* = 0.12, *p* < 0.01), subjective norms (*β* = 0.12, *p* < 0.01), and perceived behavioral control (*β* = 0.32, *p* < 0.001) were also positively associated with career intention, with perceived behavioral control showing the strongest relationship. In terms of indirect effects, work values were indirectly associated with career intention through attitude (*β* = 0.09, *p* < 0.05), subjective norms (*β* = 0.09, *p* < 0.05), and perceived behavioral control (*β* = 0.23, *p* < 0.001). The total effect of work values on career intention was 0.79 (*p* < 0.001). Compared with the direct‐effect model, in which work values were strongly associated with career intention (*β* = 0.78, *p* < 0.001), the direct effect decreased but remained statistically significant after the mediating variables were included. These findings support a partial mediation structure rather than a full mediation model.

Overall, nursing students’ career intention was associated with both the direct relationship between work values and career intention and the indirect pathways through attitude, subjective norms, and perceived behavioral control. This result suggests that work values may function not only as antecedents of TPB‐related beliefs but also as broader motivational orientations associated with nursing students’ career intention.

### 3.3. Registered Nurses’ Model Results

**FIGURE 3:**
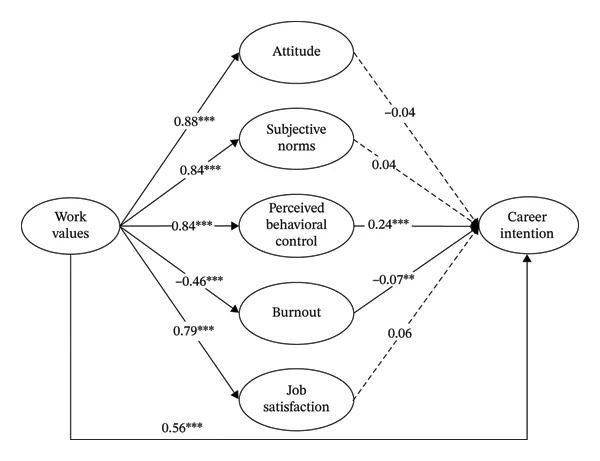
Standardized path coefficients of the registered nurse model.

To further examine the mediation structure, alternative structural models were compared. First, a direct‐effect model examining the relationship between work values and career intention was tested. Although the direct path from work values to career intention was statistically significant (*β* = 0.81, *p* < 0.001), the RMSEA was not fully satisfactory. Therefore, alternative full and partial mediation models were subsequently compared. The full mediation model and the partial mediation model were compared using *χ* ^2^ difference testing. The partial mediation model demonstrated a significantly better fit than the full mediation model (Δ*χ* ^2^ = 20.45, Δ*df* = 1, *p* < 0.001). In addition, the partial mediation model showed slightly improved fit indices, including higher CFI and TLI values and lower RMSEA and SRMR values. Specifically, CFI increased from 0.923 to 0.924, TLI increased from 0.914 to 0.915, RMSEA decreased from 0.065 to 0.064, and SRMR decreased from 0.050 to 0.049. Therefore, the partial mediation model was retained as the final model for registered nurses. The final partial mediation model demonstrated acceptable fit indices (*χ* ^2^ = 1834.25, *df* = 339, *p* < 0.001; CFI = 0.92; TLI = 0.92; RMSEA = 0.06; SRMR = 0.05). Figure 3 presents the standardized path coefficients, and Table 4 summarizes the total, direct, and indirect effects.

**TABLE 4:** Total, direct, and indirect effects in the registered nurse model.

	Estimate	S.E.	Est./S.E.
Direct effects			
Work values ⟶ attitude	0.88	0.01	72.90^∗∗∗^
Work values ⟶ subjective norms	0.84	0.01	60.34^∗∗∗^
Work values ⟶ perceived behavioral control	0.84	0.01	66.11^∗∗∗^
Work values ⟶ burnout	−0.46	0.03	−17.21^∗∗∗^
Work values ⟶ job satisfaction	0.79	0.02	45.19^∗∗∗^
Work values ⟶ career intention	0.56	0.14	4.10^∗∗∗^
Attitude ⟶ career intention	−0.04	0.07	−0.59
Subjective norms ⟶ career intention	0.04	0.06	0.76
Perceived behavioral control ⟶ career intention	0.24	0.05	4.61^∗∗∗^
Burnout ⟶ career intention	−0.07	0.02	−2.67^∗∗^
Job satisfaction ⟶ career intention	0.06	0.05	1.28
Indirect effects			
Work values ⟶ attitude ⟶ career intention	−0.04	0.08	−0.44
Work values ⟶ subjective norms ⟶ career intention	0.04	0.06	0.58
Work values ⟶ perceived behavioral control ⟶ career intention	0.20	0.06	3.62^∗∗∗^
Work values ⟶ burnout ⟶ career intention	0.03	0.01	2.40^∗^
Work values ⟶ job satisfaction ⟶ career intention	0.05	0.05	0.97
Total effect	0.84	0.02	44.60^∗∗∗^

^∗∗∗^ *p* < 0.001.

^∗∗^ *p* < 0.01.

^∗^ *p* < 0.05.

In terms of direct effects, work values had significant positive relationships with attitude (*β* = 0.88, *p* < 0.001), subjective norms (*β* = 0.84, *p* < 0.001), perceived behavioral control (*β* = 0.84, *p* < 0.001), job satisfaction (*β* = 0.79, *p* < 0.001), and career intention (*β* = 0.56, *p* < 0.001). Work values also demonstrated a significant negative relationship with burnout (*β* = −0.46, *p* < 0.001). These findings suggest that registered nurses with stronger work values tended to report more positive career attitudes, stronger perceived social expectations, higher perceived behavioral control, greater job satisfaction, lower burnout, and stronger career intention. Among the predictors of career intention, perceived behavioral control (*β* = 0.24, *p* < 0.001) and burnout (*β* = −0.07, *p* < 0.01) remained significant in the partial mediation model. However, attitude (*β* = −0.04, *p* > 0.05), subjective norms (*β* = 0.04, *p* > 0.05), and job satisfaction (*β* = 0.06, *p* > 0.05) were not significantly associated with career intention after the direct path from work values to career intention was included. In terms of indirect effects, work values were indirectly associated with career intention through perceived behavioral control (*β* = 0.20, *p* < 0.001) and burnout (*β* = 0.03, *p* < 0.05). Although the indirect effect through burnout reached statistical significance, its magnitude was relatively small and should be interpreted with caution. The indirect pathways through attitude, subjective norms, and job satisfaction were not statistically significant in the partial mediation model. The total effect of work values on career intention was 0.84 (*p* < 0.001). Compared with the direct‐effect model, in which work values were strongly associated with career intention (*β* = 0.81, *p* < 0.001), the direct effect decreased but remained statistically significant after the mediating variables were included. These findings support a partial mediation structure rather than a full mediation model.

Overall, registered nurses’ career intention was associated with both the direct relationship between work values and career intention and the indirect pathways through perceived behavioral control and burnout. This result suggests that work values may function not only as antecedents of TPB‐related beliefs and workplace psychological experiences but also as broader motivational orientations associated with nurses’ intention to remain in the profession.

### 3.4. Discussion

According to the ANOVA results, the background factors associated with nurses’ career intentions, such as age, gender, education level, and work experience, were generally consistent with findings from previous studies [3, 26, 27]. Among nursing students, statistically significant differences in career intention were observed across variables such as age, gender, nursing‐related work, or volunteer experience, and plans to pursue further education after graduation. However, some variables, such as gender (*η* ^2^ = 0.008) and nursing‐related work or volunteer experience (*η* ^2^ = 0.009), demonstrated relatively small effect sizes, suggesting limited practical significance. Students with relevant practical experience reported slightly higher career intention scores than those without such experience, although the observed effect size was small. In addition, those planning to pursue advanced studies demonstrated comparatively stronger career intentions than those who were undecided or did not intend to pursue further studies, with a moderate effect size. This finding suggests that learning opportunities and career development expectations during the educational process may be associated with stronger professional motivation among nursing students. For registered nurses, statistically significant differences in career intention were also observed across age, gender, educational attainment, years of experience, and weekly working hours. However, some variables, such as gender and weekly working hours, demonstrated relatively small effect sizes. Notably, nurses with higher education levels tended to report stronger recognition of work values and greater willingness to remain in the profession. Furthermore, nurses working 21–40 h per week reported relatively higher career intention scores than those working 41–60 h per week, although the observed effect size was small.

This study employed SEM for both nursing students and registered nurses to examine the relationships among work values, TPB constructs, workplace psychological variables, and career intention, yielding several practical and theoretical implications. First, the findings generally support the applicability of the extended TPB framework in explaining nursing career intention across both educational and clinical contexts. Among nursing students, attitude, subjective norms, and perceived behavioral control were all positively associated with career intention, while perceived behavioral control demonstrated the strongest relationship. Similarly, among registered nurses, perceived behavioral control remained significantly associated with career intention in the final partial mediation model. These findings are generally consistent with previous TPB‐related studies [11–13], suggesting that individuals’ confidence in their ability to perform nursing‐related tasks and adapt to professional challenges may represent an important factor associated with nursing career intention.

Second, work values demonstrated both direct and indirect relationships with career intention across the two groups. Among nursing students, work values were associated with career intention both directly and indirectly through attitude, subjective norms, and perceived behavioral control. In contrast, among registered nurses, indirect relationships were primarily observed through perceived behavioral control and burnout, whereas the indirect pathways through attitude and subjective norms were no longer statistically significant in the final partial mediation model. This difference may reflect variations in professional development stages between nursing students and registered nurses. For nursing students, career intention may still be strongly shaped by idealized professional attitudes and perceived social expectations from teachers, peers, and family members. Therefore, attitude and subjective norms remained important explanatory mechanisms linking work values and career intention. However, for registered nurses with actual clinical experience, career intention appeared to rely more heavily on practical evaluations of professional competence, work adaptability, and emotional sustainability within clinical settings [17–19]. In this context, perceived behavioral control may reflect nurses’ perceived capability to cope with clinical demands and continue working in the profession, whereas the influence of general attitudes and social expectations may become comparatively weaker after entering professional practice. In addition, work values demonstrated a strong direct relationship with career intention among registered nurses, suggesting that professional values may become increasingly stable motivational orientations directly associated with nurses’ career intention after entering clinical practice. Compared with the full mediation models, the partial mediation models demonstrated significantly improved fit, suggesting that work values may function not only as antecedents of TPB‐related beliefs and workplace psychological experiences but also as broader motivational orientations associated with career intention. Prior studies have reported stable relationships between personal values and TPB constructs [14–16]. The current study extends this line of research by comparing nursing students and registered nurses within the same analytical framework, thereby providing additional evidence regarding the role of work values across different professional stages in nursing.

Third, the findings for registered nurses suggest that workplace psychological experiences may play an important role in shaping career intention. Specifically, work values were negatively associated with burnout [4, 6, 27], suggesting that nurses who perceive stronger alignment between their professional beliefs and workplace environments may report lower levels of emotional exhaustion and professional burnout. In addition, burnout remained negatively associated with career intention in the final partial mediation model. These findings are generally consistent with prior studies reporting negative relationships between burnout and nurses’ retention intention [4, 17, 18, 27]. However, although the indirect effect of burnout was statistically significant, its magnitude was relatively small (*β* = 0.03) and should not be interpreted as a practically important mechanism. Therefore, the burnout pathway should be viewed as supplementary evidence of workplace psychological influences rather than a dominant explanatory process underlying nurses’ career intention. Although work values were positively associated with job satisfaction, the indirect pathway through job satisfaction was not statistically significant in the final partial mediation model after the direct path from work values to career intention was included. This finding suggests that the relationship between job satisfaction and career intention may partially overlap with broader motivational factors associated with work values.

Overall, the results of this study highlight the multifaceted psychological processes underlying career intention. Career intention appears to be associated not only with rational cognition at the level of behavioral intention but also with emotional responses and value beliefs. This finding extends previous research applying TPB in nursing contexts and provides preliminary implications for nursing education and workforce management.

## 4. Conclusions and Limitations

### 4.1. Conclusions

Grounded in the TPB, this study constructed career intention models for both nursing students and registered nurses and employed SEM to examine the underlying psychological relationships associated with nursing career intention. The findings generally support the applicability of TPB in understanding nursing career intention across both educational and clinical contexts. In both groups, work values were positively associated with the three core TPB constructs (attitude, subjective norms, and perceived behavioral control), while work values also demonstrated direct relationships with career intention in the final partial mediation models. These findings suggest that work values may function not only as antecedents of TPB‐related beliefs but also as broader motivational orientations associated with nursing career intention across different professional stages.

Furthermore, perceived behavioral control demonstrated the strongest relationship with career intention in both groups, suggesting that individuals’ confidence in their capability to perform nursing‐related tasks and adapt to professional demands may represent an important psychological factor associated with nursing career development. However, differences were observed between nursing students and registered nurses regarding the mediating mechanisms of TPB. Among nursing students, attitude, subjective norms, and perceived behavioral control were all associated with career intention. In contrast, among registered nurses, perceived behavioral control remained the primary TPB‐related pathway associated with career intention after the direct path from work values to career intention was included. This finding suggests that career intention among registered nurses may rely more heavily on practical evaluations of work adaptability and emotional sustainability within clinical settings. In addition, burnout demonstrated a negative relationship with career intention among registered nurses, whereas job satisfaction was positively associated with work values. These findings suggest that workplace psychological experiences may represent important factors associated with nurses’ willingness to remain in the profession. The present study further suggests that incorporating workplace psychological variables into TPB‐based models may contribute to a more comprehensive understanding of nursing career intention within clinical contexts.

Overall, the findings indicate that career intention may involve multiple psychological processes, including personal beliefs, social expectations, perceived behavioral control, and workplace psychological experiences. Positive work values during the educational stage may be associated with stronger professional motivation and more favorable psychological adaptation among the nursing personnel. Accordingly, nursing education and healthcare organizations may consider strengthening value‐oriented education, workplace support, and career socialization strategies as potential approaches for supporting long‐term professional retention.

### 4.2. Limitations

Several limitations of this study should be acknowledged. First, this study employed a cross‐sectional design; therefore, causal relationships among the variables cannot be definitively established. Second, the data were collected using self‐report questionnaires, which may be subject to common method bias and social desirability bias. Third, although the study simultaneously examined nursing students and registered nurses, the analyses were conducted using independent cross‐sectional samples rather than longitudinal tracking across career stages. Fourth, despite acceptable evidence of construct validity and discriminant validity, the relatively strong associations observed between work values and the TPB constructs may limit the precision with which individual indirect pathways can be interpreted. Therefore, the relative contribution of specific mediating mechanisms should be interpreted with appropriate caution. Fifth, the same samples were used for both CFA and subsequent SEM. Although this practice is common in nursing and management research, it may result in somewhat optimistic estimates of measurement model fit. Future studies are encouraged to employ split‐sample validation or independent replication to further strengthen the generalizability and robustness of the findings. In addition, future studies are encouraged to employ longitudinal or experimental designs to further examine the causal mechanisms underlying nursing career intention and to validate the long‐term relationships among work values, psychological factors, and career development outcomes.

## Author Contributions

Chung‐I. Lin: conceptualization, investigation, resources, data curation, and writing–review and editing. Chih‐Feng Lai: methodology, formal analysis, validation, data curation, interpretation of results, and writing–review and editing. Wei‐Cheng Chien: conceptualization, methodology, investigation, project administration, formal analysis, writing–original draft, writing–review and editing, and supervision. Ming‐Hsueh Tsai, Ming‐Chuan Hsieh, and Ming‐Chi Wu: investigation, resources, and writing–review and editing. Chin‐Hsiang Yen: supervision and writing–review and editing.

## Funding

This work was supported by the National Academy for Educational Research (Taiwan) under Grant No. NAER‐1141200220 and the National Science and Technology Council (Taiwan) under Grant No. NSTC 115‐2410‐H‐142‐007‐.

## Disclosure

All authors have read and approved the final version of the manuscript.

## Conflicts of Interest

The authors declare no conflicts of interest.

## Data Availability

The data that support the findings of this study are available from the corresponding author upon reasonable request.

## References

[1] Halcomb E. , Smyth E. , and McInnes S. , Job Satisfaction and Career Intentions of Registered Nurses in Primary Health Care: an Integrative Review, BMC Family Practice. (2018) 19, no. 1, 10.1186/s12875-018-0819-1. PMC6081816 30086722

[2] O′Donnell C. A. , Jabareen H. , and Watt G. C. , Practice Nurses’ Workload, Career Intentions and the Impact of Professional Isolation: a cross-sectional Survey, BMC Nursing. (2010) 9, no. 1, 10.1186/1472-6955-9-2. PMC2823612 20205777

[3] Shao J. , Tang L. , Wang X. , Qiu R. , Zhang Y. , Jia Y. , and Ye Z. , Nursing Work Environment, Value Congruence and Their Relationships with Nurses’ Work Outcomes, Journal of Nursing Management. (2018) 26, no. 8, 1091–1099, 10.1111/jonm.12641. 30221422

[4] Sibandze B. T. and Scafide K. N. , Among Nurses, How Does Education Level Impact Professional Values? A Systematic Review, International Nursing Review. (2017) 65, no. 1, 65–77, 10.1111/inr.12390. 28657173

[5] Hara Y. , Asakura K. , Yamada M. , Takada N. , and Sugiyama S. , Development and Psychometric Evaluation of the Nurses’ Work Values Scale, Nursing Open. (2023) 10, 6957–6971, 10.1002/nop2.1950. 37518936 PMC10495741

[6] Kim E. , Kim H. , and Lee T. , How are New Nurses Satisfied with Their Jobs? from the Work Value Perspective of Generations Y and Z Nurses, BMC Nursing. (2024) 23, no. 1, 10.1186/s12912-024-01928-7. PMC11032593 38643129

[7] Trede I. and Schweri J. , Work Values and Intention to Become a Registered Nurse Among Healthcare Assistants, Nurse Education Today. (2014) 34, no. 6, 948–953, 10.1016/j.nedt.2013.10.009. 24231635

[8] Zhang X. , Zhang Y. , Han J. et al., Employment Intention and Associated Factors of Nursing Graduates: A Structural Equation Model, Journal of Nursing Management. (2025) 2025, no. 1, 10.1155/jonm/7402874. PMC11972861 40223874

[9] Ajzen I. , The Theory of Planned Behavior, Organizational Behavior and Human Decision Processes. (1991) 50, no. 2, 179–211, 10.1016/0749-5978(91)90020-T.

[10] Bai X. , Wang A. , Plummer V. et al., Using the Theory of Planned Behaviour to Predict Nurses’ Intention to Undertake Dual Practice in China: a Multicentre Survey, Journal of Clinical Nursing. (2019) 28, no. 12, 2101–2110, 10.1111/jocn.14791. 30667105

[11] Côté F. , Gagnon J. , Kouffé Houme P. , Ben Abdeljelil A. , and Gagnon M.-P. , Using the Theory of Planned Behaviour to Predict Nurses’ Intention to Integrate Research Evidence into Clinical decision-making, Journal of Advanced Nursing. (2012) 68, no. 10, 2289–2298, 10.1111/j.1365-2648.2011.05922.x. 22229522

[12] Pan M. and Gao W. , Determinants of the Behavioral Intention to Use a Mobile Nursing Application by Nurses in China, BMC Health Services Research. (2021) 21, no. 1, 10.1186/s12913-021-06244-3. PMC7953719 33712012

[13] Kruse P. , Wach D. , Costa S. , and Moriano J. A. , Values Matter, Don’T They? – Combining Theory of Planned Behavior and Personal Values as Predictors of Social Entrepreneurial Intention, Journal of Social Entrepreneurship. (2018) 10, no. 2, 1–29, 10.1080/19420676.2018.1541003.

[14] Yasir N. , Mahmood N. , Mehmood H. S. , Rashid O. , and Liren A. , The Integrated Role of Personal Values and Theory of Planned Behavior to Form a Sustainable Entrepreneurial *Intention. Sustainability* , Sustainability. (2021) 13, no. 16, 10.3390/su13169249.

[15] Zhao Z. , Xue Y. , Geng L. , Xu Y. , and Meline N. N. , The Influence of Environmental Values on Consumer Intentions to Participate in agritourism—A Model to Extend TPB, Journal of Agricultural and Environmental Ethics. (2022) 35, no. 3, 10.1007/s10806-022-09881-8. PMC9360681 35965967

[16] Zhang X. , Huang H. , Zhao S. , Li D. , and Du H. , Emotional Exhaustion and Turnover Intentions Among Young ICU Nurses: a Model Based on the Job demands-resources Theory, BMC Nursing. (2025) 24, no. 1, 10.1186/s12912-025-02765-y. PMC11800589 39915810

[17] Alkhraishi M. Y. , Eivazzadeh N. , and Yeşiltaş M. , The Impact of Burnout on Turnover Intention Among Nurses: the Mediating Role of Job Satisfaction, Hacettepe Sağlık İdaresi Dergisi. (2023) 26, no. 1, 1–28.

[18] Zhao Y. , Wang H. , Sun D. et al., Job Satisfaction, Resilience and Social Support in Relation to Nurses’ Turnover Intention Based on the Theory of Planned Behaviour: a Structural Equation Modelling Approach, International Journal of Nursing Practice. (2021) 27, no. 6, 10.1111/ijn.12941. 33856093

[19] Almalki M. J. , FitzGerald G. , and Clark M. , The Relationship Between Quality of Work Life and Turnover Intention of Primary Health Care Nurses in Saudi Arabia, BMC Health Services Research. (2012) 12, no. 1, 10.1186/1472-6963-12-314. PMC3507760 22970764

[20] Delobelle P. , Rawlinson J. L. , Ntuli S. , Malatsi I. , Decock R. , and Depoorter A. M. , Job Satisfaction and Turnover Intent of Primary Healthcare Nurses in Rural South Africa: a Questionnaire Survey, Journal of Advanced Nursing. (2011) 67, no. 2, 371–383, 10.1111/j.1365-2648.2010.05496.x. 21044134

[21] Karasek R. A. , Job Demands, Job Decision Latitude, and Mental Strain: Implications for Job Redesign, Administrative Science Quarterly. (1979) 24, no. 2, 285–308, 10.2307/2392498.

[22] Herzberg F. , Work and the Nature of Man, 1966, World Publishing Company.

[23] Krejcie R. V. and Morgan D. W. , Determining Sample Size for Research Activities, Educational and Psychological Measurement. (1970) 30, no. 3, 607–610, 10.1177/001316447003000308.

[24] Dyrbye L. N. , Power D. V. , Massie F. S. et al., Factors Associated with Resilience to and Recovery from Burnout: a Prospective, multi-institutional Study of US Medical Students, Medical Education. (2010) 44, no. 10, 1016–1026, 10.1111/j.1365-2923.2010.03754.x. 20880371

[25] Leiter M. P. , Jackson N. J. , and Shaughnessy K. , Contrasting Burnout, Turnover Intention, Control, Value Congruence and Knowledge Sharing Between Baby Boomers and Generation X, Journal of Nursing Management. (2009) 17, no. 1, 100–109, 10.1111/j.1365-2834.2008.00884.x. 19166528

[26] Hu H. , Wang C. , Lan Y. , and Wu X. , Nurses’ Turnover Intention, Hope and Career Identity: the Mediating Role of Job Satisfaction, BMC Nursing. (2022) 21, no. 1, 10.1186/s12912-022-00821-5. PMC8830989 35144604

[27] Şahin M. , Bektaş G. , Nal M. , and Küçükkurt A. C. , The Mediating Role of Job Satisfaction Related to Nurse-Nurse Collaboration and Turnover Intention, BMC Nursing. (2025) 24, no. 1, 10.1186/s12912-025-02869-5. PMC11874075 40033285

